# Complete Genome Sequences of Novel Bovine T4, rv5-Like, and Dhillonviruses Effective against Escherichia coli O157

**DOI:** 10.1128/MRA.01261-20

**Published:** 2021-01-07

**Authors:** Domonkos Sváb, Linda Falgenhauer, Trinad Chakraborty, István Tóth

**Affiliations:** a Institute for Veterinary Medical Research, Centre for Agricultural Research, Budapest, Hungary; b Institute of Hygiene and Environmental Medicine, Justus Liebig University Giessen, Giessen, Germany; c German Centre for Infection Research, Site Giessen-Marburg-Langen, Giessen, Germany; d Institute for Medical Microbiology, Justus Liebig University Giessen, Giessen, Germany; Queens College

## Abstract

Enterohemorrhagic Escherichia coli (EHEC) isolates of serotype O157:H7 are serious foodborne zoonotic pathogens and prime targets for biocontrol using bacteriophages. We report on the complete genome sequences of 11 novel lytic bacteriophages, representing three viral genera, isolated from cattle in Hungary that target E. coli O157 strains.

## ANNOUNCEMENT

The role of bacteriophages as alternative antimicrobial agents has gained renewed interest in the past years (1). Enterohemorrhagic Escherichia coli (EHEC) strains, particularly of the O157:H7 serotype, are serious foodborne zoonotic pathogens, and several phages which show effective lysis of these have previously been characterized (2, 3). Presently, there are authorized products for use against EHEC as biocontrol in foodstuffs (EcoShield [4]). Nevertheless, a strategy for characterizing new phage isolates that target the eradication of these pathogens from various environments remains prudent.

Here, we present the genome sequences of 11 new lytic bacteriophages (Table 1). Nine phages originated from fecal samples of healthy cattle, the main reservoir of EHEC (5), were isolated from dairy and meat-producing farms in Hungary, using the EHEC O157:H7 strain Sakai for propagation as described earlier (3). Two of the phages were isolated from a sample taken from the cattle farm environment using the same protocol. Briefly, after precultivation of the samples in 10 volumes of tryptic soy broth with bile salts overnight at 37°C under aerobic conditions, the bacterium-free filtrate was spotted onto layered Luria-Bertani agar plates with the propagating strain. All phages were active against the reference strains EHEC O157:H7 Sakai (6) and EDL933 (7); the T4-like and rV5-like phages lysed several bovine E. coli O157 strains representing EHEC, enteropathogenic E. coli (EPEC), and atypical pathotypes isolated earlier in Hungary, as well (8).

**TABLE 1:** List of phage genomes announced in the current study with their main characteristics

Phage	GenBank accession no.	Raw read data accession no.	Total no. of reads	Sequence length (nt)^*a*^	Coverage (×)	Topology	Termini	Predicted genus
vb_EcoM_bov9_1	MT884006	SRR13132432	10,168,954	166,440	494.836	Linear	Fixed	*Tequatrovirus*
vb_EcoM_bov10K1^*b*^	MT884007	SRR13132431	9,085,150	166,441	78.3038	Linear	Fixed	*Tequatrovirus*
vb_EcoM_bov10K2^*b*^	MT884008	SRR13132431	9,085,150	135,960	2,261.21	Linear	Circular permutated	*Vequintavirus*
vb_EcoM_bov11CS3	MT884009	SRR13132430	9,449,636	135,960	1,724.25	Linear	Circular permutated	*Vequintavirus*
vb_EcoM_bov22_2^*c*^	MT884010	SRR13132429	3,442,978	135,961	983.78	Linear	Circular permutated	*Vequintavirus*
vb_EcoM_bov25_3^*d*^	MT884011	SRR13132428	11,006,422	135,961	3,327.32	Linear	Circular permutated	*Vequintavirus*
vb_EcoS_bov11C2	MT884012	SRR13132430	9,449,636	44,612	3,830.02	Linear	Circular permutated	*Dhillonvirus*
vb_EcoS_bov16_1	MT884013	SRR13132427	15,936,284	44,745	527.902	Linear	Circular permutated	*Dhillonvirus*
vb_EcoS_bov22_1^*c*^	MT884014	SRR13132429	3,442,978	44,612	71.37	Linear	Circular permutated	*Dhillonvirus*
vb_EcoS_bov25_1D^*d*^	MT884015	SRR13132428	11,006,422	44,747	374.845	Linear	Fixed	*Dhillonvirus*
vB_EcoM_bov15_1	MT951623	SRR13132426	1,856,660	44,700	607.948	Linear	Circular permutated	*Dhillonvirus*

a nt, nucleotides.

b These phages were isolated from the same environmental sample.

c These phages were isolated from the same bovine fecal sample.

d These phages were isolated from the same bovine fecal sample.

Phage DNA from active lytic stocks was isolated using the phenol-chloroform method (9) and concentrated with the DNA Clean and Concentrator kit (Zymo Research, CA, USA) according to the manufacturer’s instructions. Genomic DNA sequencing libraries were prepared using the Nextera XT kit (Illumina, Eindhoven, Netherlands). Sequencing was performed using the NextSeq midoutput reagent kit v2.5 (2 × 150 bp) on an Illumina NextSeq 500 machine. Coverage was between 71× and 3,830×, with an average of 1,298× (Table 1). Quality control and clipping of the reads were performed within the framework of the ASA^3^P pipeline (10). ASA^3^P quality control/read clipping includes the programs FastQC (https://github.com/s-andrews/FastQC), FastQ Screen (https://www.bioinformatics.babraham.ac.uk/projects/fastq_screen), and Trimmomatic (11). The types of genome termini were determined using PhageTerm with default parameters (12). All phages depicted a linear topology (Table 1). Assembly was performed with SPAdes 3.10.1 (13). Homology searches were conducted against GenBank with the megaBLAST algorithm, available at the NCBI website; annotation was performed with the RAST server using the RASTtk algorithm with default parameters (14). The sequencing showed that some stocks contained multiple phages; therefore, purification of single plaques followed, and PCR for marker genes with primers specifically designed (data not shown) was used to separate single-phage stocks. Phages originating from the same sample are marked as such in Table 1.

Phages vb_EcoM_bov9_1 and vb_EcoM_bov10K1 proved to be T4-like phages, representing the *Tequatrovirus* genus within the *Myoviridae* family, with 95% average nucleotide identity to the type phage T4 assessed by BLAST (GenBank accession number MT984581.1), genome sizes of 166,440 and 166,441 bp, respectively, and a GC content of 35.4%. Phages vb_EcoM_bov10K2, vb_EcoM_bov11CS3, vb_EcoM_bov22_2, and vb_EcoM_bov25_3 are rV5-like phages belonging to the *Vequintavirus* genus of the *Vequintavirinae* subfamily, having a >96% nucleotide identity to the type phage rV5 (DQ832317.1). Their genome sizes were between 135,960 and 135,961 bp, with a 43.7% GC content. Phages vb_EcoS_bov11C2, vb_EcoS_bov15_1, vb_EcoS_bov16_1, vb_EcoS_bov22_1, and vb_EcoS_bov25_1D represent HK578-like phages, officially the *Dhillonvirus* genus from the *Siphoviridae* family, with 86% genome coverage and >90% nucleotide identity to phage HK578 (JQ086375.1). Their genome sizes were between 44,612 and 44,747 bp, and their GC content was 54.5%.

Phages within the same genus were very uniform. The T4-like phages differed in only a 1-bp gap. Of the rV5-like phages, vb_EcoM_bov10K2, vb_EcoM_bov11CS3, and vb_EcoM_bov25_3 were 100% identical except for a 1-bp gap, while vb_EcoM_bov22_2 differed from them in only 2 single nucleotide polymorphisms (SNPs). The dhillonviruses differed at most by 11 SNPs, with a gap of 30 or 89 bp. The genome of vb_EcoS_bov22_1 was assembled as a partial genome sequence, with a different start position compared to the other dhillonviruses.

### Data availability.

The nucleotide sequences of the phages are deposited in GenBank under the accession numbers MT884006 through MT884015 and MT951623.
